# Quality of knee osteoarthritis care in the Netherlands: a survey on the perspective of people with osteoarthritis

**DOI:** 10.1186/s12913-022-08014-1

**Published:** 2022-05-12

**Authors:** J. M. H. Oomen, Y. A. S. Peters, C. H. van den Ende, H. J. Schers, W. J. J. Assendelft, J. E. Vriezekolk, S. Koëter

**Affiliations:** 1 Department of Research and Innovation, Sint Maartenskliniek, PO Box 9011, 6500 GM Nijmegen, the Netherlands; 2 Department of Primary and Community Care, Radboudumc, PO Box 9101, 6500 HB Nijmegen, the Netherlands; 3 Orthopedics, Canisius Wilhelmina Hospital, Weg door Jonkerbos 100, 6532 SZ Nijmegen, the Netherlands

**Keywords:** Osteoarthritis, Quality indicator, Knee, Hip, Primary care, General practitioner

## Abstract

**Background:**

Quality indicators (QIs) are used to monitor quality of care and adherence to osteoarthritis (OA) standards of care. Patient reported QIs can identify the most important gaps in quality of care and the most vulnerable patient groups. The aim of this study was to capture the perspective of people with knee OA (KOA) in the Netherlands on the quality of care received, and explore determinants related to lower achievement rates.

**Methods:**

We sent an online survey to all members of The Dutch Knee Panel (*n* = 622) of the Sint Maartenskliniek Nijmegen, the Netherlands between September and October 2019. The survey consisted of a slightly adapted version of the “OsteoArthritis Quality Indicator” (OA-QI) questionnaire (18 items; yes, no, N/A); a rating of quality of KOA care on a 10-point scale; a question on whether or not one wanted to see change in the care for KOA; and an open-ended question asking recommendations for improvement of OA care. Furthermore, sociodemographic and disease related characteristics were collected. Pass rates for separate QIs and pass rates on patient level were calculated by dividing the number of times the indicator was achieved by the number of eligible persons for that particular indicator.

**Results:**

A total of 434 participants (70%) completed the survey. The mean (SD) pass rate (those answering “Yes”) for separate QIs was 49% (20%); ranging from 15% for receiving referral for weight reduction to 75% for patient education on how to manage knee OA. The mean (SD) pass rate on patient level was 52% (23%). Presence of OA in other joints, comorbidities, and having a knee replacement were associated with higher pass rates. On average, a score of 6.5 (1.6) was given for the quality of care received, and the majority of respondents (59%) wanted change in the care for KOA. Of 231 recommendations made, most often mentioned were the need for tailoring of care (14%), more education (13%), and more empathy and support from healthcare providers (12%).

**Conclusion:**

This study found patients are only moderately satisfied with the OA care received, and showed substantial gaps between perceived quality of care for OA and internationally accepted standards. Future research should focus on the underlying reasons and provide strategies to bridge these gaps.

**Supplementary Information:**

The online version contains supplementary material available at 10.1186/s12913-022-08014-1.

## Background

Osteoarthritis (OA) is one of the most prevalent forms of musculoskeletal disability worldwide and is expected to become the most common condition in the Netherlands as of 2040 [1]. OA causes structural joint changes that can lead to pain, fatigue, limitations in daily activities, and loss in quality of life [2–7]. Knee osteoarthritis (KOA) is the most prevalent form of OA [8]; the lifetime risk for developing symptomatic KOA being 44.7% [9]. Although there are no curative treatment options for OA, multiple effective treatments are available to reduce symptoms and limitations in activities based on a stepped-care approach [10] that combines lifestyle advice, physical/exercise and dietary therapy if applicable, and pain medication. When non-surgical options are unsuccessful, joint replacement is an option. Internationally, conservative treatment options are underused in the management of KOA [11–21], while timely usage of these treatment modalities is advocated and may prevent untimely surgery.

To monitor quality of care and adherence to standards of care, quality indicators (QIs) based on medical records or health care provider questionnaires can be used. In recent years, patient-reported QIs have become more popular, as they may include aspects of care which simply cannot be extracted from a medical record. Previous research on patient-reported quality of care for KOA shows a wide variability in reported usage of conservative treatment options for OA across countries, with overall achievement rates of QIs ranging from 22 to 57% [22–32]. Although achievement of most individual QIs differed strongly, referral to weight loss programs for eligible patients structurally yielded the lowest percentage in achievement and delivery of information about the importance of exercise the highest percentage in achievement across countries. Previous research identified age [24, 27, 33, 34], level of education [27, 34], gender [28], OA severity [27, 33], and contact with multiple health care providers [24] as determinants of achievement rates at the patient level. Age might be a significant factor in the interplay of OA achievement rates as the number of comorbidities is likely to rise with age, making it more difficult for health care providers to sort out which disease(s) to address [26, 34]. However, a recent study by Østerås et al. [23] could neither confirm the association of age with QI achievement, nor the association of gender, education, and severity.

Research concerning KOA patient self-reported QIs is still scarce. To our knowledge, no studies concerning KOA patient-reported QIs in the Netherlands have been published to date. However, mapping patients’ experiences is a valuable and important step in the process of improving quality of care. The use of patient-reported QIs provides a better understanding whether the recommendations actually transpire in practice and enable us to identify possible areas for improvement in current OA care. Thus, the main aim of this study was to capture the perspective of people with KOA in the Netherlands on the quality of care received, and to explore determinants related to lower achievement rates.

## Methods

### Study design

A cross-sectional online survey among people with KOA was conducted in the Netherlands. This paper was written according to the Strengthening the Reporting of Observational Studies in Epidemiology (STROBE) statement [35] and the Checklist for Reporting Results of Internet E-Surveys (CHERRIES) [36].

### The Dutch Knee Panel

Recruitment took place through the ongoing Dutch Knee Panel, founded in February 2019 by the Department of Rheumatology, Sint Maartenskliniek, Nijmegen, the Netherlands, a specialized hospital in disabilities of posture and movement. Respondents were recruited via social media and via informational material within the hospital. People could enter their contact details via a website (www.kniepanel.nl) after which they would receive a baseline questionnaire. The panel consists of people with a self-reported clinical diagnosis or with suspicion of KOA. Inclusion criteria for the panel consisted of the clinical classification criteria for knee OA set up by the American College of Rheumatology (clinically diagnosed with KOA or experiencing knee pain for most days of the month, over a period of at least three consecutive months), Dutch language proficient in word and writing, living in the Netherlands, and aged 40 years or over. As people with OA often suffer from multiple diseases and causes of OA differ widely, no specific exclusion criteria have been set. The Dutch Knee Panel was established for research purposes. All members gave permission to be invited for research studies, as well as the re-use and merge of data from multiple research studies. The Dutch medical research ethics committee of Arnhem-Nijmegen waived ethical approval since the panel is not subject to the medical research involving human subjects act (file number 2018–4832).

### Data collection

For this study, all members of The Dutch Knee Panel (*N* = 622) were invited between September and October 2019 to complete our online survey. Castor Electronic Data Capture (EDC) was used to distribute the survey online. Each participant received an e-mail containing information on the study including aim and a unique hyperlink, and the survey was locked after completion. Participation in the survey was voluntary and no incentives were given. After two weeks, a reminder was sent to those who did not respond to the initial survey request. The survey took 10 to 15 min to complete and included open-ended as well as close-ended questions. The questionnaire consisted of 11 pages, and each page consisted of between 1 and 9 items. No randomization of items or adaptive questioning techniques have been used. Participants were required to answer each question in order to continue, and when applicable items were provided with a non-response option. Respondents were able to review and change their answers via a “Back” button.

### Baseline measurement

Participants were obliged to complete a questionnaire to initially enrol in the panel, which formed the baseline characteristics of the present study. Time difference between this baseline questionnaire and the questionnaire of the present study was 6.2 (SD 1.5) months on average. In this panel introduction questionnaire, participants were asked for sociodemographic characteristics in terms of gender (male/female), age (years), education (secondary education or lower (i.e. elementary, high school, and technical or vocational training); higher education (i.e. research universities and universities of applied sciences)), and height (cm) and weight (kg). Furthermore, self-reported health status was assessed using the EQ-5D consisting of a short descriptive system questionnaire (EQ-5D-5L)[37] and a visual analogue scale (EQ-VAS). The individual health levels were converted into the Dutch EQ-5D index values. The visual analogue scale (VAS) ranged from 0 (“worst imaginable health”) to 100 (“best imaginable health”). Severity of OA symptoms was assessed through duration of symptoms (less than one year, between one and five years, more than 5 years), and the presence of OA symptoms (“Have you received a diagnosis from a doctor or other care provider for osteoarthritis in joints other than the knee?”) in the following joint groups: hands, wrists, elbows, shoulders, neck, back, hips, ankles, feet. Daily functioning was assessed with the Dutch version of the Knee Osteoarthritis Outcome Score (KOOS)[38]. The subscale concerning activities of daily living (ADL) consist of 17 items with 5-point Likert scales addressing the extent of problems (no problems – extreme problems) experienced in daily living over the past week. Furthermore, the number of comorbidities was assessed by means of a list of 20 predetermined categories (including “other, namely…”). Lastly, participants were asked to indicate the level of KOA related pain (VAS).

### OsteoArthritis Quality Indicator (OA-QI) questionnaire

To assess the quality of OA care, a slightly modified version of the OsteoArthritis Quality Indicator (OA-QI) questionnaire was used. The original OsteoArthritis Quality Indicator questionnaire is validated to measure the quality of primary care for OA [39], and comprises of 17 questions related to patient education and information, regular provider assessments, referrals, and pharmacologic treatment [26]. The OA-QI questionnaire was translated into Dutch, and slightly modified to represent all facets of the Dutch guidelines for OA care by three researchers (YP/JV/CE) working within rheumatology who have experience with questionnaire design. Three patient representatives commented on ease of completion and any ambiguous words or difficult to understand statements by means of a pre-test. The final version of the questionnaire contained all 17 items of the original OA-QI, which were either identical or contained subtle changes in wording to items in the validated original (Additional file 1). One additional item was included derived from the Dutch guidelines or OA care (“Have you discussed a follow-up appointment with your healthcare professional to check up on your OA symptoms and treatment?”). A complete overview of the Dutch questionnaire can be found in additional file 2. The final questionnaire consisted of 18 items addressing whether or not a participant had received certain types of treatment for KOA (“Yes”, “No”, and either “I do not remember” or “Not applicable”). This Dutch version of the OA-QI questionnaire showed acceptable internal consistency (α = 0.79).

### Additional measurements

Participants were asked whether they had received a knee arthroplasty (yes/no; left, right, both) and if so, when (in the past two years, longer than two years ago). Furthermore, healthcare use concerning KOA was assessed through questions whether or not the patient contacted health care professional(s) (general practitioner, physical therapist, orthopaedic surgeon, rheumatologist, other) for their KOA complaints in the past year (yes/no). Participants additionally rated the overall quality of care they had received for their KOA on a 10-point scale (1: very poor—10: excellent), were asked if they wanted to see change in the care for KOA(yes/no), and if so, to make recommendations for OA healthcare improvement via an open-ended question.

### Statistical analysis

Only participants who fully completed the survey were included in the analyses. Continuous variables were reported as means with standard deviations or median with IQR where applicable, and categorical variables as percentages. Guided by the original OA-QI study [26], QI pass rates were calculated for each QI separately for the study sample as a whole, where the numerator represents the number of patients reporting “yes” (indicator passed) and the denominator represents the number of eligible persons for that particular indicator (those reporting “yes” or “no”, i.e. excluding “not applicable”). Then, the mean (SD) pass rates for separate QIs was calculated by dividing the number of times the indicator was achieved by the number of eligible persons for that particular indicator. Correspondingly, the mean (SD) of pass rates on patient level were calculated by dividing the total number of QIs passed by the total number of QIs for which they were eligible [26]. Determinants of pass rates on patient level were assessed through linear regression analyses (Stata 13, StataCorp). Furthermore, the initial responses to the open-ended questions were reviewed by two of the researchers (YP, JO) to construct a coding scheme based on major categories that emerged by means of data-driven coding. To determine interrater reliability, 29 of the 141 responses were randomly selected to be blindly coded by the two independent raters. After consultation on the coding and interpretation, agreement was made resulting in an interrater reliability of *κ* = 0.87, *p* < 0.001. The two researchers then independently coded the data. Subsequently, a top-5 was formed based on the number of times a recommendation was made in one of the categories.

## Results

### Participants

All 622 panel members were invited to participate in the study. Of those, 171 did not respond to the invitation or the reminder (27.5%), and 17 participants did not complete the survey (2.7%), resulting in 434 participants for analyses (response rate: 69.8%). Comparison of patient and disease characteristics between responders and non-responders showed that responders are likely to be representative for the Dutch Knee Panel (data not shown). Participants’ characteristics are presented in Table 1. Participants were mostly female with an average age of 63 years old. The population consisted of people with moderate OA on average based on a mean scoring on daily functioning of 64 out of 100 (SD 18), more than half of the participants (54%) reported to have OA in other joints besides the knee, and a small minority (13%) had received a knee arthroplasty. The majority of participants (56%) had been experiencing complaints for more than five years and almost three quarters suffered from comorbidities (70%). Further validation showed 98.2% of respondents (*n* = 426) met at least three of the ACR criteria.

**Table 1:** Characteristics of participants (*n* = 434)

Characteristic	
Female, n (%)	305 (70)
Age (in years), mean (SD)	63.3 (8.8)
Education, n (%)	
• Secondary education or lower	223 (51)
• Higher education	211 (49)
BMI, mean [kg/m^2^], (SD)	27.8 (4.8)
EQ-5D-5L index score, mean (SD)	0.71 (0.1)
EQ-VAS (0–100), mean (SD)	70.7 (14.8)
Daily functioning (KOOS^a^; 0–100), mean (SD)	63.5 (18)
Pain VAS (0–10), mean (SD)	5.4 (2.1)
Duration of symptoms, n (%)	
• Less than one year	30 (7)
• Between one and five years	163 (38)
• More than five years	241 (56)
Presence of OA in other joints, n (%)	232 (54)
Number of other joint groups with OA (1–9), median (IQR)	2 (1–8)
Knee replacement, n (%)	57 (13)
Presence of comorbidities, n (%)	306 (70.5)
Number of comorbidities (1–20), median (IQR)	2 (1–6)

*BMI* Body Mass Index, *VAS* Visual Analogue Scale, *OA* Osteoarthritis; a: The Knee Injury and Osteoarthritis Outcome Score (KOOS) is a percentage score with 0 representing extreme problems and 100 representing no problems

### Use of healthcare services

Most participants (*n* = 339, 78%) had had contact with healthcare professional(s) for their KOA complaints in the past year; most often with an exercise or physical therapist (53%), a general practitioner (44%) or an orthopaedist/orthopaedic surgeon (42%). Of these participants, 110 (25.4%) contacted two healthcare professionals, and 116 (26.7%) contacted three to five health care professionals in the past year.

### Quality indicators

The mean (SD) pass rate of individual QIs achieved was 49% (20%), ranging from 15%—74% (Table 2), with referral for weight reduction least often achieved, and receiving advice on managing/living with osteoarthritis most often. The mean (SD) pass rate on patient level was 52% (23%). The QI questions can be found in their entirety in additional file 1.

**Table 2:** Patient-reported pass rates for all 18 quality indicators (*n* = 434)

Quality indicator		Eligible^a^ n	Achieved^b^ n (%)
1	Received information about KOA	417	310 (74.3)
2	Received information about treatment options	424	252 (59.4)
3	Received advice about selfcare of knee complaints	421	313 (74.4)
4	Received practical support in selfcare of knee complaints	421	253 (60.1)
5	Received information or advice about exercising and sports	426	306 (71.8)
6	Offered referral for support in exercising and sports	423	211 (49.9)
7	Advised to lose weight	303	111 (36.6)
8	Offered referral for weight loss support	247	37 (15.0)
9	Received assessment on limitations in daily activities	301	89 (29.6)
10	Received assessment on need for walking aid	273	65 (23.8)
11	Received assessment on need for support with daily activities	284	52 (18.3)
12	Received assessment on pain complaints	426	240 (56.3)
13	Advised to take paracetamol as first choice	424	312 (73.6)
14	Offered stronger pain killer(s)	421	132 (31.4)
15	Offered anti-inflammatory painkiller (NSAIDs)	422	229 (54.3)
16	Offered injection in knee	429	239 (55.7)
17	Discussed knee replacement surgery	362	223 (61.6)
18	Offered follow-up appointment	427	144 (33.7)

^a^Excluding “not applicable” and “do not remember”; ^b^Checked ‘Yes’; *OA* Osteoarthritis

### Determinants

Significant associations were found for participants’ summary QI pass rates with self-reported presence of OA in other joints, presence of comorbidities, having a knee replacement, and having had contact with several (between two and four) healthcare professional in the past year. No other significant associations were found (Table 3).

**Table 3:** Characteristics as determinants of QI summary pass rates (*n* = 434)

Characteristic	Coef. = *b*	95% CI
Gender		
• Male (ref)		
• Female	3.8	[-0.9; 8.5]
Age	-0.2	[-0.4; 0.1]
Education		
• Secondary education or lower (ref)		
• Higher education	0.9	[-3.4; 5.2]
BMI	-0.4	[-0.8; 0.1]
Daily functioning^a^	-0.1	[-0.2; 0.1]
Pain VAS	0.3	[-0.7; 1.3]
Pain for longer than 3 months	3.6	[-10.1; 17.3]
Number of days in pain over the past month	0.1	[-0.2; 0.3]
Duration of symptoms		
• < 1 year (ref)		
• 1–5 years	-1.4	[-10.3; 7.5]
• > 5 years	1.5	[-7.2; 10.1]
Presence of OA in other joints	**6.2**	[2.0; 10.5]
Number of other joints affected by OA	0.7	[-0.9; 2.2]
Knee replacement	**10.7**	[4.4; 17.0]
Presence of comorbidities	**5.3**	[0.6; 10.0]
Number of comorbidities	-0.5	[-2.3; 1.4]
Contacted healthcare professional in past year		
• None (ref)		
• One	5.5	[-0.4; 11.5]
• Two	**13.4**	[7.5; 19.4]
• Three or more	**19.6**	[13.8; 25.5]

Significant determinants in **bold**, *Ref* reference, *BMI* Body Mass Index, *OA* Osteoarthritis, a: higher scores equal lower QI summary pass rates

### Perceived quality of healthcare

Participants rated the OA quality of care with a 6.5 (SD 1.6) on average. A total of 254 (59%) participants indicated that they wanted to see improvement in OA care, of whom 141 made one or more suggestions (231 suggestions in total). Table 4 shows an overview of the top 5 most mentioned recommendations. Other topics were the need for support in the self-management of OA (5.2%), healthcare providers (HCPs) not treating OA as a serious condition (4.8%), increasing the ease of access to aids and information (4.3%), increasing HCPs’ knowledge in treatment options of OA and a better alignment of care between HCPs (3.5%), and increasing ease of access to care and reducing waiting times (3.0%). Some exemplary recommendations made by the participants: *“It would be an improvement if different professionals work together in one team”, “More tailored care, no two OA complaints are the same”, “Recognition that living with so much pain sometimes seems useless”, “Faster diagnosis, more explanation of cause, course and treatment methods”. A complete overview of the different topics presented via the open-ended question can be found in additional file* 3.

**Table 4:** Top 5 most mentioned recommendations (*n* = 231)

#	Recommendation	n (%)
1	HCPs should provide tailor-made advice in line with symptoms	33 (14.3)
2	More education on OA	30 (13.0)
3	More empathy and support from HCPs	28 (12.1)
4	More choice in treatment options	14 (6.1)
5	HCPs should pay more attention to the personal circumstances of the individual and to pain complaints	13 (5.6)

## Discussion

### General findings

Our study shows that only about half of the QIs for OA care are achieved and that patients are moderately satisfied with the quality of OA care. Achievement of individual QIs differed strongly, with referral for weight reduction least often achieved and receiving advice on managing/living with osteoarthritis most often. Moreover, none of the QIs exceed a 75% pass rate. Associations were found between summary QI pass rates and presence of OA in other joints, presence of comorbidities and having a knee replacement. In addition, having had contact with several healthcare professionals in the past year was also related to QI summary pass rates. Lastly, the majority of participants wanted to see change in the care for KOA in terms of more information and education, and better alignment and tailoring of care.

### Comparison with literature

The overall QI pass rate in the present study was similar to the patient-reported OA-QI achievement rates in primary healthcare in other western countries, ranging from 47 to 57 percent [22–27]. On the basis of these observations one might conclude that the quality of care for KOA in the Netherlands is satisfactory. However, comparing QI pass rates should be done with caution because the study samples, settings, and methods may differ, as well as healthcare systems and access to OA care across countries. For instance, in the Netherlands the general practitioner acts as a gatekeeper for referral to secondary care services such as an orthopaedic surgeon, whereas other services such as dietary or physical therapy are freely accessible. Only in about half of the European countries GPs act as gatekeeper to specialist care. In other countries, such as for instance Greece and Switzerland, there is a strong emphasis on hospital care [40].

Our results suggest that little progress has been made in the past four years in the improvement of quality of care in the Netherlands as our results are comparable with the 2016 report on rheumatology care in the Netherlands [41], which concluded that although individuals with OA are generally satisfied with the quality of OA care, there was still room for improvement. In line with previous studies, our findings reveal that core elements of treatment are suboptimally utilized in daily practice. Pass rates of QIs reflecting provision of education and support in self-management performed relatively well. However, one out of four participants did not receive adequate education, indicating substantial room for improvement. The latter finding is underscored by the need for more and tailored information as expressed in free text. Noteworthy, only half of the participants indicated that referral for exercise therapy and/or weight reduction was offered as a treatment option, whereas both treatment options are an essential part of Dutch guideline recommendations [42]. Healthcare providers might need better education on availability and efficacy of physical exercise and weight loss programs for the management of OA [43, 44]. They should furthermore develop more trust in the efficacy of these programs in terms of patient’s efforts as well as their service providers [20, 45, 46].

In contrast to other studies, we found no relationship between QI summary pass rates and demographic variables such as age [24, 27, 33, 34], gender [27, 34], and educational level [27, 34]. Our finding suggests QIs cannot identify most vulnerable patient groups by demographic variables, as treatment modalities seem to be used for patients of all ages, regardless of their gender or educational level. We did find a relation between QI summary pass rates and the presence of comorbidities, in line with several other studies [26, 34]. This might be due to more frequent visits with the healthcare professional, which offers more opportunity for treatment. People who have moderate OA complaints without other comorbidities might not visit a healthcare professional on a regular basis, believing there simply is no cure for their osteoarthritis. Another explanation is the lack of follow-up appointments offered by the professional, as results show a low pass rate on this particular quality indicator. As found in a previous study [24], contact with healthcare professionals was strongly related to higher QI pass rates, suggesting patient who do not visit their healthcare professional on a regular basis might miss out on appropriate care and information. In addition, in line with previous research [27, 33] we found that having had a knee prosthetic and the presence of OA symptoms in other joints were strongly related with higher QI pass rates. These findings also underline the need for more continuous guidance of patients with hip and knee OA.

### Strengths and limitations

The present study was the first to make use of KOA patient-reported QIs in the Netherlands. Mapping patients’ experiences is a valuable and important step in the process of improving quality of care. The use of patient-reported QIs provides a better understanding whether the recommendations actually transpire in practice and enable us to identify possible areas for improvement in current OA care. In addition, the Dutch Knee Panel, used in the present study for patient recruitment, consists of people with OA symptoms in the knee from all over the country, making the sample more diverse than most studies, often centered in a specific region. Sociodemographic and disease related characteristics corresponded well to the general population of Dutch primary care, and members have proven to be highly willing with response rates nearing at least 70% for this and previous studies.

The present study also has several limitations. A first limitation of the present study could be that using panel members is prone to volunteer bias, as participants of the panel might possess different psychosocial and clinical characteristics from the target population [47]. For instance, research show that women are more likely to volunteer for studies than males [48]. A second limitation could be the self-reporting of OA by the patient instead of a clinical diagnosis. However, the accuracy of self-reported OA has been found to be acceptable (sensitivity of 0.75 and specificity of 0.89) for large scale studies [49], and the Dutch Knee Panel has the advantage of offering such large sample size. In addition, quality of care was also assessed by means of self-report, which might be more prone to accuracy and recall bias [50]. Nonetheless, quality of care is in the ‘eye of the beholder’ and patients are the ones at the receiving end of care. Moreover, most participants had had fairly recent contact with a healthcare professional (4 out of 5 in the past year) in the present study, making recollections retrieved by study participants regarding events or experiences from the past less prone to bias. Self-report could, however, have resulted in an under- or overestimation of certain QIs. Further analysis of QI 7 (“Have you been advised to lose weight?”) and QI 8 (“If you are overweight, have you been offered a referral for weight loss support (e.g. to a dietician or a weight loss group)?”) showed that around 1 out of 5 overweight respondents did not consider themselves to be overweight while they actually were in terms of BMI scores (data not shown). As the majority were not severely overweight (BMI < 30/m2), it seems they do not perceive themselves as overweight and might not have received this classification by their healthcare provider. When including these answers as ‘No’, QI pass rates lower from 37 to 32% for QI 7, and from 15 to 12% for QI 8. As a result, it is very likely that the presented pass rates for QI 7 and QI 8 are an overestimation. In terms of QI 18 ("Have you discussed a follow-up appointment with your healthcare professional to checkup on your OA symptoms and treatment?"), reimbursement policies of healthcare costs could have played a role in follow-up visits, as patients have to pay obligatory deductible excess for visits to a medical specialist, and require additional assurance for physio- and dietary therapy. However, the focus of QI 18 is only on whether it was discussed, not whether respondents have actually had a follow-up. Further analysis shows there is not much difference between consulted healthcare professionals and the discussion of a follow-up visit. A total of 39% of respondents have discussed a follow-up with their physiotherapist, 43% with their rheumatologist, 45% with their GP, and 53% with their orthopedic surgeon. Furthermore, the average time difference of 6 months between the baseline questionnaire and survey could have resulted in imprecise results, in particular with regard to the association between daily functioning and perceived quality of care. Lastly, a Dutch version of the OA-QI questionnaire is used for the present study which has not yet been validated. Small changes have been made to the original validated questionnaire for a better fit with the Dutch OA healthcare guidelines, of which measurement and psychometric properties have not been tested. However, the contribution from patient representatives as well as experts within the field ensured that the resulting OA-QI incorporated issues relevant to patients with OA, written in a language that patients found easy to understand. Given the similarity between the construct and wording of the questionnaires, for the current Dutch version of the questionnaire similar measurement properties as the validated original version of the OA-QI from Norway were assumed, as did a subsequent version from the UK [51]. However, two findings in the present study (data not shown) reinforce construct validity of our Dutch version of the OA-QI questionnaire: 1) Overall rating for the quality of care on a scale from 1 to 10 was positively correlated with QI summary pass rates—the higher the rating, the higher the pass rate; 2) Participants who wanted to see change in the care they had received scored significantly lower in QI summary pass rates. Nonetheless, the assumption that the measurement properties of the questionnaires are similar may need further exploration and conducting a full validation study is recommended.

### Recommendations

Based on our findings we recommend that healthcare professionals should aim for higher adherence to standards of care and guideline recommendations, as it may improve patients’ outcomes and postpone the need for total joint replacements. Special attention to weight loss services is needed. Alignment of care between GPs and other healthcare providers, as also often mentioned in the recommendations by patients, might improve referral to such services. The recommendations made via the open-ended question in this study offered a first glance on areas of improvement in KOA care as experienced by patients. It is recommended to further investigate these patient recommendations as certain themes seem to recur more often than not. Lastly, preparing the survey we noticed that the common questionnaires on osteoarthritis quality indicators often do not include the assessment of follow-up appointments in patient-provider consultations (QI 18 of the present study). Considering the patients’ need for guidance in the self-management process of their OA, we strongly advise to include such a question in the future.

## Conclusion

Our study shows that on average less than half of the individual QIs for OA care are achieved, and none of the QIs exceed a 75% pass rate, indicating substantial room for improvement. Furthermore, individual patients are only moderately satisfied with the quality of OA care. The majority of participants wanted to see change in the care for KOA in terms of more information and education, and better alignment and tailoring of care. As the prevalence of OA is expected to further increase over the coming years, adherence to standards of care and guideline recommendations is becoming increasingly important. Future efforts should involve joint initiatives consisting of multifaceted interventions including both patients and healthcare professionals to improve the quality of OA care and implementation of guidelines.

## Supplementary Information

**Additional file 1. **Overview of OA-QI questionnaire.

**Additional file 2. **Dutch OA-QI questionnaire.

**Additional file 3. **Recommendations through open-ended question.

## Data Availability

The datasets used and/or analysed during the current study are available from the corresponding author on reasonable request.

## Abbreviations

OA: Osteoarthritis
KOA: Knee OA
QI: Quality Indicator
STROBE: Strengthening the Reporting of Observational Studies in Epidemiology
CHERRIES: Checklist for Reporting Results of Internet E-Surveys
VAS: Visual Analogue Scale
KOOS: Knee Osteoarthritis Outcome Score
OA-QI: OsteoArthritis Quality Indicator
SD: Standard Deviation
IQR: Inter Quartile Range
Ref: Reference
BMI: Body Mass Index
NIVEL: Netherlands Institute for Health Services Research
