# Supplementary material for: Quality of knee osteoarthritis care in the Netherlands: a survey on the perspective of people with osteoarthritis
Source: BMC Health Serv Res. 2022 May 12;22:631. doi: 10.1186/s12913-022-08014-1 (PMC9097380; doi:10.1186/s12913-022-08014-1)
Supplement: Supplementary file 1 — Additional file 1. Overview of OA-QI questionnaire. [file 12913_2022_8014_MOESM1_ESM.docx]

Additional file 1. Overview of OA-QI questionnaire

| # | Original OA-QI (Østerås et al., 2013) | Dutch OA-QI |
| --- | --- | --- |
| 1 | Have you been given information about how the disease usually develops over time? | Have you received information about knee osteoarthritis from a healthcare professional? |
| 2 | Have you been given information about different treatment alternatives? | Have you received information from a healthcare professional about the different treatment options for your knee OA? |
| 3 | Have you been given information about how you can live with the disease? | Have you received advice from a healthcare professional about what you can do about your knee complaints yourself? |
| 4 | Have you been given information about how you can change your lifestyle? | Have you received practical support from a healthcare professional in what you can do about your knee complaints yourself? |
| 5 | Have you been given information about the importance of physical activity and exercise? | Have you received information or advice from a healthcare professional about exercise and sports to help with your knee problems? |
| 6 | Have you been referred to someone who can advise you about physical activity and exercise? (e.g., a physiotherapist) | Have you been offered a referral to a healthcare professional who can advise you on (muscle strengthening) exercises and physical activity? |
| 7 | If you are overweight, have you been advised to lose weight? | Have you been advised to lose weight? |
| 8 | If you are overweight, have you been referred to someone who can help you to lose weight? | If you are overweight, have you been offered a referral for weight loss support (e.g. to a dietician or a weight loss group)? |
| 9 | If you have had problems related to daily activities, have these problems been assessed by health personnel in the past year? | If you have trouble with daily activities because of your knee OA, have they been assessed? |
| 10 | If you have problems with walking, has your need for a walking aid been assessed? (e.g., stick, crutch, or walker) | If you have difficulty walking, has a healthcare professional assessed the need for an aid (e.g. a stick, crutch or walker)? |
| 11 | If you have problems related to other daily activities, has your need for different appliances and aids been assessed? (e.g., splints, assistive technology for cooking or personal hygiene, a special chair) | If you have difficulty with daily activities due to your knee osteoarthritis, has a healthcare professional assessed the need for aids (e.g. a splint, an adapted chair, aids for cooking or personal hygiene)? |
| 12 | If you have pain, has it been assessed in the past year? | Has the degree of pain you experience in your knee been assessed by a healthcare professional? |
| 13 | If you have pain, was acetaminophen the first medicine that was recommended for your osteoarthritic pain? | Was paracetamol the first medication you have been advised to take by a healthcare professional? |
| 14 | If you have prolonged severe pain, which is not relieved sufficiently by paracetamol, have you been offered stronger pain killers? (e.g., co‐proxamol, co‐dydramol, tramadol, co‐codamol, dihydrocodeine, codeine) | Have you been offered a stronger painkiller than paracetamol (e.g. Co-proxamol, Codydramol, Tramadol, Co-codamol, Dihydrocodeine, Codeine)? |
| 15 | If you are taking antiinflammatory drugs, have you been given information about the effects and possible side effects of this medicine? (e.g., ibuprofen, Nurofen, Brufen, diclofenac, Voltarol, naproxen, Naprosyn, Celebrex) | Have you been offered an anti-inflammatory painkiller (e.g. Ibuprofen, Nurofen, Brufen, Diclofenac, Voltarol, Naproxen, Naprosyn, Celebrex)? |
| 16 | If you have experienced an acute deterioration of your symptoms, has a corticosteroid injection been considered? | Has an injection been offered in the knee? |
| 17 | If you are severely troubled by your osteoarthritis, and exercise and medicine do not help, have you been referred and assessed for an operation? (e.g., joint replacement) | Has joint replacement surgery (“a new knee”) been discussed in the case of severe knee osteoarthritis? |
| 18 |  | Have you discussed a follow-up appointment with your healthcare professional to checkup on your OA symptoms and treatment? |
