# Supplementary material for: Quality of knee osteoarthritis care in the Netherlands: a survey on the perspective of people with osteoarthritis
Source: BMC Health Serv Res. 2022 May 12;22:631. doi: 10.1186/s12913-022-08014-1 (PMC9097380; doi:10.1186/s12913-022-08014-1)
Supplement: Supplementary file 2 — Additional file 2. Dutch OA-QI questionnaire. [file 12913_2022_8014_MOESM2_ESM.docx]

**Behandeling van knieartrose**

We willen graag van u weten welke behandelingen u heeft ontvangen voor uw knieartrose. Met een ‘zorgverlener’ bedoelen we bijvoorbeeld uw eigen huisarts, oefen- of fysiotherapeut, orthopedisch chirurg of reumatoloog. Kruis het juiste vak aan.

|  | **Ja** | **Nee** | **Weet ik niet meer** |
| --- | --- | --- | --- |
| 1. Heeft u informatie ontvangen over knieartrose van een zorgverlener? |  |  |  |
| 1. Heeft u informatie ontvangen van een zorgverlener over de verschillende behandelopties voor uw knieartrose? |  |  |  |
| 1. Heeft u advies gekregen van een zorgverlener over wat u zelf kunt doen aan uw knieklachten? |  |  |  |
| 1. Heeft u ondersteuning ontvangen van een zorgverlener bij wat u zelf kunt doen aan uw knieklachten? |  |  |  |
| 1. Heeft u informatie of advies gekregen van een zorgverlener over lichaamsbeweging en sport om u te helpen bij uw knieklachten? |  |  |  |
| 1. Is u een verwijzing aangeboden naar een zorgverlener die u kan adviseren over (spierversterkende) oefeningen en beweegactiviteiten ? |  |  |  |
|  | **Ja** | **Nee** | **Geen overgewicht** |
| 1. Heeft u het advies gekregen om af te vallen? |  |  |  |
| 1. Als u overgewicht heeft, is u een verwijzing aangeboden ter ondersteuning bij het afvallen (b.v. naar een diëtist of een afvalgroep)? |  |  |  |
|  | **Ja** | **Nee** | **Geen moeite met lopen** |
| 1. Als u moeite heeft met lopen, is door een zorgverlener bekeken of u een hulpmiddel (b.v. een stok, kruk of rollator) nodig heeft? |  |  |  |
|  | **Ja** | **Nee** | **Geen moeite met dagelijkse activiteiten** |
| 1. Als u moeite heeft met dagelijkse activiteiten door uw knieartrose, zijn die in kaart gebracht? |  |  |  |
| 1. Als u moeite heeft met dagelijkse activiteiten door uw knieartrose, is door een zorgverlener bekeken of u hulpmiddelen (b.v. een spalk, een aangepaste stoel, hulpmiddelen bij het koken of de persoonlijke hygiëne) nodig heeft? |  |  |  |
|  | **Ja** | **Nee** | **Geen pijn aan mijn knie** |
| 1. Is de mate van pijn aan uw knie door een zorgverlener beoordeeld? |  |  |  |
| 1. Is u, door een zorgverlener, geadviseerd om paracetamol te nemen als eerste pijnstiller? |  |  |  |
| 1. Is u een sterkere pijnstiller dan paracetamol (b.v. Co-proxamol, Codydramol, Tramadol, Co-codamol, Dihydrocodeine, Codeine) aangeboden? |  |  |  |
| 1. Is u een ontstekingsremmende pijnstiller (b.v. Ibuprofen, Nurofen, Brufen, Diclofenac, Voltarol, Naproxen, Naprosyn, Celebrex) aangeboden? |  |  |  |
|  | **Ja** | **Nee** | **Weet ik niet meer** |
| 1. Is een injectie in de knie aangeboden? |  |  |  |
|  | **Ja** | **Nee** | **Ik heb geen ernstige last** |
| 1. Is bij ernstige last van knieartrose, een gewricht vervangende operatie (“een nieuwe knie”) besproken? |  |  |  |
|  | **Ja** | **Nee** | **Weet ik niet meer** |
| 1. Heeft u met uw zorgverlener besproken wanneer u terugkomt voor een controle van uw knieartrose en behandeling? |  |  |  |
