# Supplementary material for: Quality of knee osteoarthritis care in the Netherlands: a survey on the perspective of people with osteoarthritis
Source: BMC Health Serv Res. 2022 May 12;22:631. doi: 10.1186/s12913-022-08014-1 (PMC9097380; doi:10.1186/s12913-022-08014-1)
Supplement: Supplementary file 3 — Additional file 3. Recommendations through open-ended question. [file 12913_2022_8014_MOESM3_ESM.docx]

Additional file 3. Recommendations through open-ended question

| Type of recommendation made | N |
| --- | --- |
| Tailored advice/in line with someone's symptoms | 33 |
| Expand awareness | 30 |
| More empathy/support from an expert | 28 |
| Expanding treatment options | 14 |
| Attention to personal situation | 13 |
| More attention to pain complaints | 13 |
| Provide support in the self-management process | 12 |
| take seriously / treatment | 11 |
| Facilitate access to tools and information | 10 |
| Expert caregivers | 8 |
| Multidisciplinary care | 8 |
| Preference for surgery | 8 |
| Facilitate access to care | 7 |
| Shorten waiting times | 7 |
| Reimbursement in care package | 5 |
| Provide clear information | 4 |
| Expanding scientific research | 4 |
| Provide consistent information | 3 |
| More attention to fatigue complaints | 3 |
| Improve aftercare | 3 |
| Provide current information | 2 |
| More attention to weight loss | 2 |
| More attention for prevention | 2 |
| Information in understandable language | 1 |
